# Supplementary material for: Blood lactate dynamics in awake and anaesthetized mice after intraperitoneal and subcutaneous injections of lactate—sex matters
Source: PeerJ. 2020 Jan 6;8:e8328. doi: 10.7717/peerj.8328 (PMC6951280; doi:10.7717/peerj.8328)
Supplement: Table S1 [file peerj-08-8328-s001.pdf]

Supplementary Table 1. Population distribution within treatment groups.

Isoflurane	Injection	Number of wildtype/transgenic mice	Number of males/females	Average age (weeks)
-	Lactate, IP	3 / 3	2 / 4	11.9
	Lactate, SC	2 / 4	3 / 3	12.1
	PBS, IP	3 / 2	3 / 2	13.5
	PBS, SC	4 / 2	3 / 3	13.5
+	Lactate, IP	3 / 3	3 / 3	17.0
	Lactate, SC	2 / 4	3 / 3	15.5
	PBS, IP	2 / 4	3 / 3	15.0
	PBS, SC	2 / 2 (1 n/a) †	3 / 2	15.5

IP – intraperitoneal injection
SC – subcutaneous injection
PBS – phosphate-buffered saline
† 1 n/a refers to one mouse for which genotyping was not performed
