# Supplementary material for: Blood lactate dynamics in awake and anaesthetized mice after intraperitoneal and subcutaneous injections of lactate—sex matters
Source: PeerJ. 2020 Jan 6;8:e8328. doi: 10.7717/peerj.8328 (PMC6951280; doi:10.7717/peerj.8328)
Supplement: Table S2 [file peerj-08-8328-s002.pdf]

Supplementary Table 2. Average, minimum and maximum time of blood sampling.

Timepoint	Average (sec..)	Average (in min.)	Minimum (sec.)	Maximum (sec.)
1	285	5	220	440
2	756	13	700	980
3	2196	37	2090	2360
4	3605	60	3590	3625
